# Visualizing and Evaluating Finger Movement Using Combined Acceleration and Contact-Force Sensors: A Proof-of-Concept Study

**DOI:** 10.3390/s21051918

**Published:** 2021-03-09

**Authors:** Hitomi Oigawa, Yoshiro Musha, Youhei Ishimine, Sumito Kinjo, Yuya Takesue, Hideyuki Negoro, Tomohiro Umeda

**Affiliations:** 1 Department of MBT, Graduate School of Medicine, Nara Medical University, Nara 634-8521, Japan; hitomi-oigawa@naramed-u.ac.jp; 2 Toho University Ohashi Medical Center, Department of Orthopedic Surgery, Toho University, Tokyo 153-8515, Japan; musha@oha.toho-u.ac.jp (Y.M.); gogozettaigokaku@yahoo.co.jp (Y.I.); sumitokinjo@yahoo.co.jp (S.K.); yuya19840921@yahoo.co.jp (Y.T.); 3 Brigham and Women’s Hospital and Harvard Medical School, Boston, MA 02115, USA; negoroh-tky@umin.ac.jp; 4 MBT Institute, Nara Medical University, Nara 634-8521, Japan

**Keywords:** healthcare, wearable sensor, acceleration, strain, 10-s grip and release, grip strength

## Abstract

The 10-s grip and release is a method to evaluate hand dexterity. Current evaluations only visually determine the presence or absence of a disability, but experienced physicians may also make other diagnoses. In this study, we investigated a method for evaluating hand movement function by acquiring and analyzing fingertip data during a 10-s grip and release using a wearable sensor that can measure triaxial acceleration and strain. The subjects were two healthy females. The analysis was performed on the x-, y-, and z-axis data, and absolute acceleration and contact force of all fingertips. We calculated the variability of the data, the number of grip and release, the frequency response, and each finger’s correlation. Experiments with some grip-and-release patterns have resulted in different characteristics for each. It was suggested that this could be expressed in radar charts to intuitively know the state of grip and release. Contact-force data of each finger were found to be useful for understanding the characteristics of grip and release and improving the accuracy of calculating the number of times to grip and release. Frequency analysis suggests that knowing the periodicity of grip and release can detect unnatural grip and release and tremor states. The correlations between the fingers allow us to consider the finger’s grip-and-release characteristics, considering the hand’s anatomy. By taking these factors into account, it is thought that the 10-s grip-and-release test could give us a new value by objectively assessing the motor functions of the hands other than the number of times of grip and release.

## 1. Introduction

With the development of medicine, life expectancy is increasing around the world [1]. In particular, Japan is called a super-aging society, and the aging rate is expected to increase in the future [2]. The difference between the average life expectancy and healthy life expectancy (the period time when a person can live without restrictions on daily life due to health problems) in Japan is 8.5 years for men and 10.2 years for women [3]. This difference is the length of time where some care is needed, and we need to close this gap by extending healthy life expectancy.

Nursing care’s principal purpose is to assist the patient’s motor function [4,5,6,7,8]. The Barthel index, a method for assessing activities of daily living (ADLs), consists of 10 items: feeding, bathing, grooming, dressing, bowels, bladder, toilet use, transfers, mobility, and stairs [9]. All the items are related to the dexterity in the hands. It has also been reported that hand dexterity is associated with cognitive function [10,11], and grip strength is associated with dementia [12,13], diabetes [14], stroke [15], aging [16], carpometacarpal osteoarthritis [17], carpal tunnel syndrome [18], and cervical spondylotic myelopathy (CSM) [19]. It is necessary to establish a system with which a person grasps the condition of his or her hands daily and maintains the health status of the hands to detect and treat diseases at an early stage.

Currently, the box and block test (BBT) [20], the Purdue pegboard test [21], and the 10-s grip and release [22] are used to evaluate the operation of the hand motor. It is necessary to visit a medical institution for assessment and to assess these motor functions. Then, we let a specialist evaluate it. However, research on quantification and hand movements is conducted using sensors and devices. 

Recently, sensors and Internet of Things (IoT) technologies have enabled various data collection, bidirectional communication, data management, and evaluation in the cloud. In the medical field, the use of such technologies is increasing. Moreover, data fusion and integration from various medical devices are improving, and mobile technologies are accelerating. Patient-generated health data using mobile health are attracting [23,24,25]. 

The types of sensors and devices were used to capture hand movements with nine-axis inertial measurement unit (IMU), acceleration, pressure and bending sensors, electromyography, magnetoencephalography (MEG), functional magnetic resonance imaging (fMRI), leap motion, virtual reality (VR), motion capture, and tablets [26,27,28,29,30,31,32,33,34,35,36,37,38,39,40,41,42,43,44].

In the nine-axis IMU study, a glove combined with a novel stretchable substrate material and nine degrees of flexible inertial sensors was developed to detect joint movements [26]. They suggested that the glove was useful for tracking the rehabilitation progress of patients with arthritis. The acceleration studies compared the hand-grasping-actions characteristics of Parkinson’s disease with those of healthy subjects [27] and assessed and automatically scored the finger-tapping movements in patients with idiopathic Parkinson’s disease and atypical parkinsonism [28]. They developed a glove that acquires the pressure applied to the fingertips with the importance of grip strength [29] in a study using a pressure sensor. There was also research using the bend sensor in terms of the development of a glove that can be worn on the hand and fingers [30]. Having used electromyography, they assessed multiple sclerosis [31] and movements such as ADLs and the Purdue pegboard test in combination with motion capture [32]. In a study using magnetoencephalography and functional magnetic resonance imaging, data on differences of movements of fingers between healthy subjects and stroke patients were obtained to assess the recovery of sensory and motor function in stroke patients [33]. A study using leap motion showed the motion data from the simple test for evaluating hand function [34] and evaluated movements after stroke rehabilitation [35]. In a VR study, a conventional BBT was developed on a VR and validated [36]. Using motion capture, they quantitatively characterized the opening and closing of the hand in healthy subjects [37] and stroke patients. They assessed the movement of the upper limb as it moved an object [38]. A tablet-based study collected data on accuracy, pressure, and tracing duration when healthy subjects and CSM patients traced a sine wave displayed on a tablet device at a comfortable pace. Based on the data, they assessed the CSM patient’s hand dexterity [39]. Moreover, other studies have combined a smartwatch with a flexible hand-mounted sensor (with built-in triaxial acceleration and gyroscopes) to detect tremor symptoms in Parkinson’s disease [40], an infrared imaging device [41], a rehabilitation robot [42], and other devices for evaluating hand dexterity [43,44].

These sensors and devices are expected to make it possible to assess hand motor function in all diseases. The study of the quantification method by measuring the movement with a sensor is effective to eliminate the variability in evaluation depending on the observer’s skill and experience, which is a problem. In particular, the acquisition and results of 10-s grip-and-release data could add new value.

The 10-s grip and release counts how many times the hand can be opened and closed in 10 s. It can be easily administered at home and was featured on Japanese TV health programs as a guide for medical checkups and a way to check your health. If the number of times is 20 or less, the hand is considered a dexterity disorder (primarily CSM) [22]. However, previous studies have argued whether a single threshold of 20 times is sufficient to make a correct assessment because there were gender and age differences [45,46]. Moreover, because each part of the hand moves differently with nerves, muscles, and cervical vertebrae of the upper extremity, each finger’s movements were abnormal even though the frequency is regular when focusing on the finger movements. Experienced physicians and other observers can evaluate the specific area and disease from the 10-s grip and release based on the above, but it is difficult for the average person.

In a previous study, the 10-s grip and release was lengthened to 15 s and recorded with a digital camera for validation with JOA scores [47,48,49] to assist doctors and others in diagnosis. In addition, they studied a method for quantitative and automatic measurement of hand movement function by leap motion, because the method’s practicality was reduced by checking the hand movement after taking photographs [50,51]. In a study by sensors to assess 10-s grip-and-release movements, we used leap motion to detect cervical myelopathy by machine learning (random forest) [52]. These results suggest that it is useful for evaluating a 10-s grip and release’s behavior by using a sensor. However, only the presence or absence of disabilities can be determined at this stage; therefore, it is impossible to identify the affected area or what kind of disease is likely to be present. 

The purpose of this study was to objectively evaluate the 10-s test in addition to the threshold of 20 times, not only by doctors and other medical professionals but also by the public. In this study, we focused on the grip-and-release motion during the 10-s grip and release and acquired the result by using acceleration and strain sensors. We extracted and analyzed features from the data obtained and investigated whether it was possible to evaluate hand elaboration movements in addition to the number of grip and release by visualizing them. The reason for using acceleration and strain sensors instead of leap motion is to consider the speed and trajectory of the 10-s grip and release and the movement and force of each finger. Since strength is related to grip, this data increases the number of diseases. Considering the above, we conducted experiments with HapLog, which is equipped with acceleration and strain sensors. Section 2 described the sensors. We also describe the processing and evaluation methods of the acquired data. Based on this, Section 3 and Section 4 present evaluation experiments and discussion.

## 2. Materials and Methods

### 2.1. HapLog

**Figure 1:**
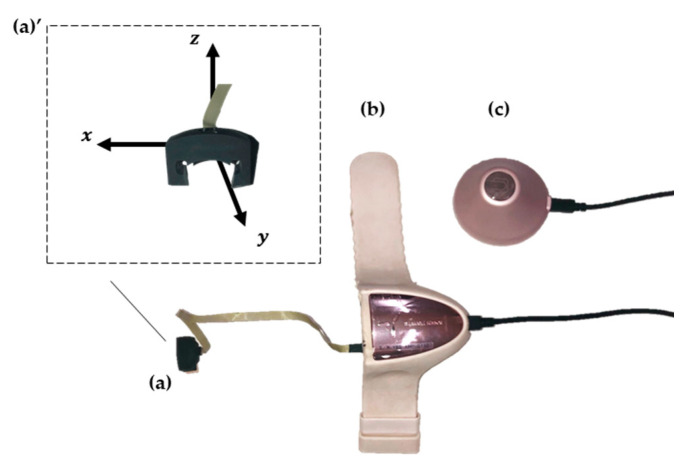
HapLog’s structure (**a**) sensor, (**a**)’ front of the sensor: the left and right accelerations were x-axis accelerations, front and back accelerations were y-axis accelerations, and up and down accelerations were z-axis accelerations, (**b**) bangle-type connector, and (**c**) calibration unit.

We used a wearable sensor, the contact-force sensor HapLog (Kato Tech Co., Ltd., Kyoto, Japan), for acquiring data [53]. The HapLog consisted of a sensor worn on the fingertip, a bangle-type connector to the connected sensor, and a calibration unit (Figure 1).

It was necessary to connect the connector and the calibration unit to a Personal Computer (PC) via USB and to run the HapLog software to use them.

The sensor attached to the finger was covered on the nail side. There were five different sizes of covers (11, 12, 14, 16 and 18 mm) to fit each nail. A triaxial accelerometer and a strain sensor were mounted on the cover. With the cover-up, the left and right accelerations were the x-axis, the front and back accelerations were the y-axis, and the top and bottom accelerations were the z-axis. The strain sensor detected strains by transmitting finger deformation to the cover. The sensor’s sampling frequency was selectable from 1 to 1000 Hz, and the acceleration range was selectable from 2 to 8 G. 

We connected up to three sensors to the bangle-shaped connector. The digital signals of measurement were displayed in real-time using HapLog software, and information on up to three sensors is saved in a single CSV file. The file includes the following information (Table 1): 

The information includes the HapLog used, the timestamp at the start of the measurement, and memo fields. In the sensor information, the position in the connector, the name of the target data(finger contact force, absolute acceleration, raw contact-force data, x-axis acceleration, y-axis acceleration, z-axis acceleration, and the mark), the unit of the target data (N for finger contact force, G for absolute acceleration, x-axis acceleration, y-axis acceleration, and z-axis acceleration, με for raw contact-force data, and dots for marks), the number of target data, the maximum value, and the minimum value of the target data were displayed. The raw contact-force data pointed to the strain, and the finger contact force was the data converted from the raw contact-force data by the calibration unit. The following equation can express the x-axis acceleration ($x_{i}$), y-axis acceleration ($y_{i}$), and z-axis acceleration ($z_{i}$) at the ith time.

(1) $${\mathrm{Absolute}\;\mathrm{Acceleration}}_{i}=\sqrt{{x_{i}}^{2}+{y_{i}}^{2}+{z_{i}}^{2}}$$

The mark indicates that its button was clicked during measurement in the operation screen of the HapLog software. In the measurement data, we measure the time from zero (starting time), and the value of each target data at that time is recorded in the row of the time, and each column is a time series of the target data. Two decimal places show finger contact force, absolute acceleration, x-axis acceleration, y-axis acceleration, and z-axis acceleration.

### 2.2. Measurement

**Figure 2:**
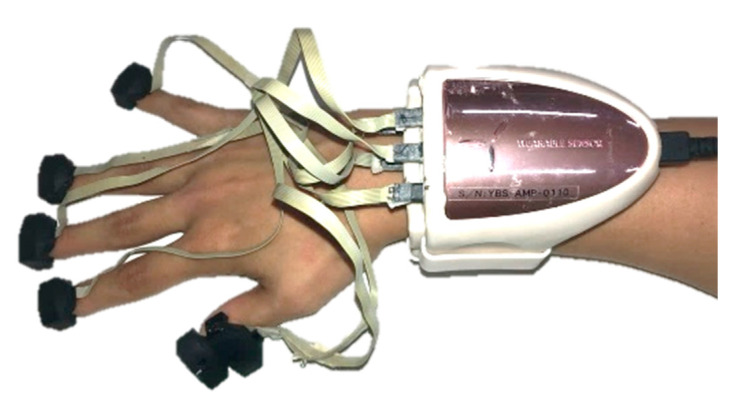
Mounting of sensors. Two HapLogs were used, and a sensor of each HapLog was attached to the thumb.

In this study, we used two HapLogs and two computers to measure five fingers: thumb, index, middle, ring, and little fingers. However, the use of an unlinked device may cause a gap in the measurement start time; therefore, we attached one of the sensors to the thumb of each HapLog and corrected the difference by preprocessing, as shown in Section 2.3 (Figure 2).

The subjects were two members of our laboratory. They were women in their 20 s and 40 s, and both were healthy. We explained the study and the experiment to the subjects and obtained their written consent to participate in the experiment.

The subjects wore the HapLog and gripped and released their hands under the following conditions.

1. Freely
2. As soon as possible
3. Slowly
4. Irregularly
5. Shivering
6. Without moving thumb as much as possible
7. Without moving the index finger as much as possible
8. Without moving the middle finger as much as possible
9. Without moving the ring finger as much as possible
10. Without moving little finger as much as possible

The grip-and-release motions were performed by the left and right hands for 20 s each. The sampling frequency was 200 Hz and the acceleration range was set to 4 G. Condition 1, which was standard, and the subject did grip and release her hand in a natural state without any instruction. As the same method, the grip-and-release actions were performed as quickly as possible in condition 2. From condition 3 onward, we set up the grip-and-release patterns for the difficulty for disease, symptoms, or other reasons. Video recording was also performed during the measurements.

### 2.3. Preprocessing

We corrected the gap in the measurement start time. Specifically, we obtained time series data of the absolute acceleration of the thumb from two sensors attached to the thumb and calculated multiple cross-correlation coefficients. We found the cross-correlation coefficient  r for the number of data  n by the following equation.

(2) $$r=\frac{\sum_{i=1}^{n}\left(x_{i}-\bar{x}\right)\left(y_{i}-\bar{y}\right)}{\sqrt{\sum_{i=1}^{n}{\left(x_{i}-\bar{x}\right)}^{2}}\sqrt{\sum_{i=1}^{n}{\left(y_{i}-\bar{y}\right)}^{2}}}$$

x is the thumb data of one HapLog and y is the thumb data of the other HapLog, with $\bar{x}$ and $\bar{y}$ being the mean of each. The numerator of the equation represents the covariance of the thumb data calculated from each HapLog. The denominator represents the standard deviation of the thumb data calculated from each HapLog. In this study, we shifted the 4 s time-series data of one HapLog by one point from 0 to 2 s, and calculated the cross-correlation function with the time-series data of the other HapLog and found the maximum value. According to the above parameters, 400 cross-correlation coefficients with n=800 data were calculated from the time-series data acquired at a sampling frequency of 200 Hz, and the point with the highest value was selected as the reference point for the start of the measurement.

We obtained 10 s of data (2048 data points). A stable grip-and-release motion occurred later than 5 s after the newly established start point by correcting the misalignment. The data were obtained after 5 s from the start to get a stable grip-and-release motion not the grip-and-release motion of the start. The reason for the 10 s was to evaluate the 10-s grip and release. The number of data was set to 2048 because it needed to be a power of 2 for the frequency analysis in the 2.4 evaluation. We defined the data to be evaluated as absolute acceleration, raw contact-force data, x-axis acceleration, y-axis acceleration, and z-axis acceleration. The reason for excluding the finger contact force from the measurement data in the table was that the values were based on HapLog’s algorithm using a calibration unit. We also did not operate the mark during the measurement, and for this reason, it was excluded. 

### 2.4. Evaluation

The following items were obtained from the data and used in the evaluation.

- Data variation
- Number of times to grip and release
- Frequency characteristics
- Correlation of each finger

In terms of data variability, we calculated the standard deviation of each finger and each item of time series data (absolute acceleration, x-axis acceleration, y-axis acceleration, z-axis acceleration, and raw contact-force data) for each subject and grip-and-release condition. We compared the standard deviations of conditions 1 to 10 by finger and item in the two groups of significant difference of the left and right hands. We compared the standard deviation of conditions 1 to 10 for each subject by finger and item for significant between-subject differences. To intuitively understand the characteristics of grip and release, we visualized the variability of each finger’s data on each item in a radar chart. A study on communicating the value of healthcare using radar charts reported that radar charts, which incorporated data on patient outcomes and costs, allowed for comprehensive and collaborative discussions [54]. This study also aims to comprehensively capture the data of all fingers of one hand and extract their characteristics by using radar charts.

The number of grip and release was the same as in the 10-s grip and release. We compared the results with the number of grip and release calculated by visual inspection and evaluated the matching rate. The target data were each finger and item of time series data (absolute acceleration, x-axis acceleration, y-axis acceleration, z-axis acceleration, and raw contact-force data) and their summation over the same time series. However, before adding up, we found the maximum and minimum values for each finger’s same item. We normalized to 0–1 based on those values. The combinations to be added were $2^{25}\left(=33,554,432\right)$. The number of grip and release was calculated by dividing the maximum and minimum values of the time series data by two. The number of times the data exceeded or fell below the threshold was divided by two (rounded up to the nearest whole number). The number of grip and release was counted, and the accuracy for all combinations was calculated.

We performed frequency analysis on each finger and item of time-series data (absolute acceleration, x-axis acceleration, y-axis acceleration, z-axis acceleration, and raw contact-force data) for each subject and grip-and-release condition. We used the fast Fourier transform for frequency analysis. To evaluate the periodicity of grip and release, we calculated the power of the most powerful frequency divided by the total power from the frequency analysis results.

In the correlation of each finger, we calculated the cross-correlation coefficients between each finger and each item of time-series data (absolute acceleration, x-axis acceleration, y-axis acceleration, z-axis acceleration, and raw contact-force data) for each finger, and grip-and-release condition. The cross-correlation coefficients were the same as (2) in Section 2.3. However, x was the time-series data of a certain item on a certain finger,  y was the time series data of the same item on a finger different from x, and the means of each were $\bar{x}$ and $\bar{y}$. The number of data was *n* = 2048 for 10 s.

## 3. Results

Based on the 2.4 evaluation, the experiment is as follows: in presenting the results, we label each item in each finger with the alphabetic combinations shown in Table 2 (e.g., absolute acceleration of the thumb, Ta).

In addition, A is a female in her 20 s, and B is a female in her 40 s.

### 3.1. Data Variation

A paired *t*-test was used for the left and right significant difference test. For the between-subjects significant difference test, we used the Student *t*-test, the Welch t-test, and the Wilcoxon signed-rank test, as a result of the Shapiro–Wilk test and the *F*-test. The table below showed the significant differences (Table 3).

The significant difference between the left and right sides was found in the x-axis acceleration of the ring finger, which was common to the subjects. There were more significantly different items for subject A than for subject B. Significant differences between subjects were common to the left and right sides: raw contact-force data of the thumb, raw z-axis acceleration, and contact-force data of the index finger, x-axis acceleration of the middle finger, and x-axis acceleration of the ring finger. There was a particularly large difference in the raw contact-force data.

**Figure 3:**
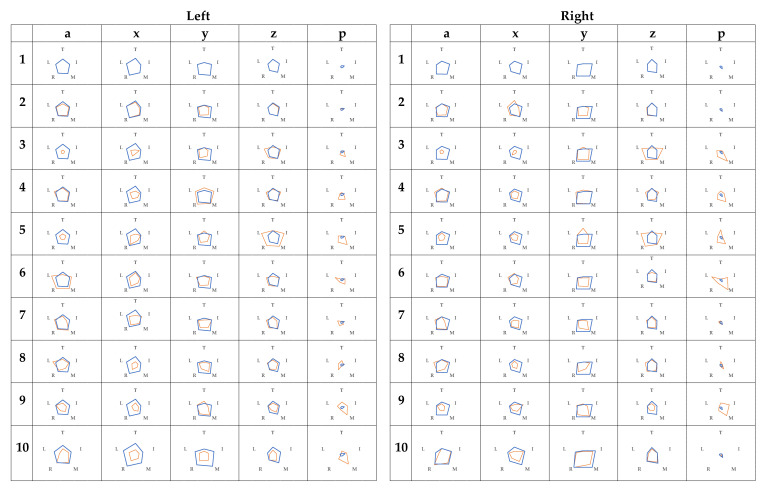
Radar charts of A (Top: thumb, clockwise, index, middle, ring, and little fingers).

**Figure 4:**
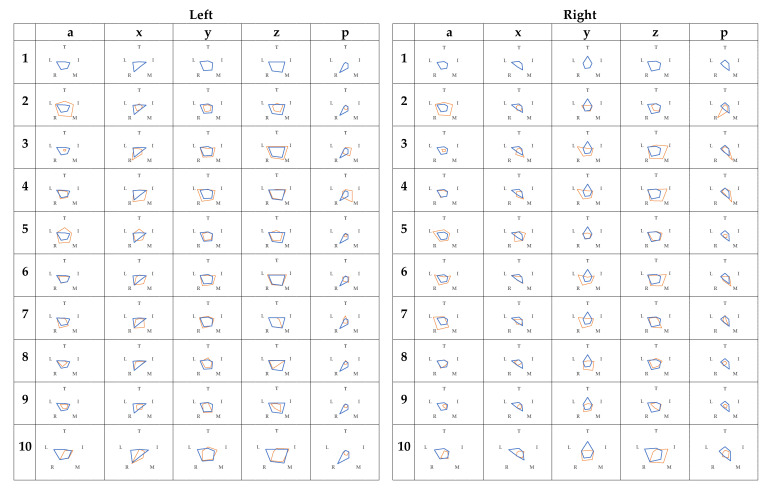
Radar charts of B (Top: thumb, clockwise, index, middle, ring, and little fingers).

Figure 3 showed the variability of each finger’s data for each item in subject A, represented by radar charts for each grip-and-release condition. Condition 1 is shown in blue, and conditions from 2 onward are shown in orange to overlap with condition 1. Similarly, the radar charts for subject B are shown in Figure 4.

The variation of absolute acceleration in condition 3 was small for all fingers in both subjects A and B when condition 1 was used as a reference. However, the raw contact-force data of the middle finger were large. In addition, the variability of the z-axis acceleration outside of the thumb tended to be large. In condition 4, there was no change in the variation of absolute acceleration. The variation of absolute acceleration of the finger was smaller than that of the first condition, and the variation of the z-axis acceleration tended to be smaller than that of the first condition.

In addition to the above, subject A tended to show a large variation in the x-axis acceleration in both the left and right sides in condition 1. In condition 2, the variation of the y-axis acceleration tended to be small for both sides, but the overall variation did not change much. In condition 5, the variation in the overall absolute acceleration was small, and the variation in the z-axis acceleration of the index, ring, and little fingers was large. Concerning subject B, there was a large variance in the raw contact-force data of condition 1 ring finger in the left hand. Moreover, the variation of the x-axis acceleration of the middle finger tended to be small. In condition 2, the variation of absolute acceleration was larger. In condition 5, contrary to A, there was a large variation in the overall absolute acceleration.

**Figure 5:**
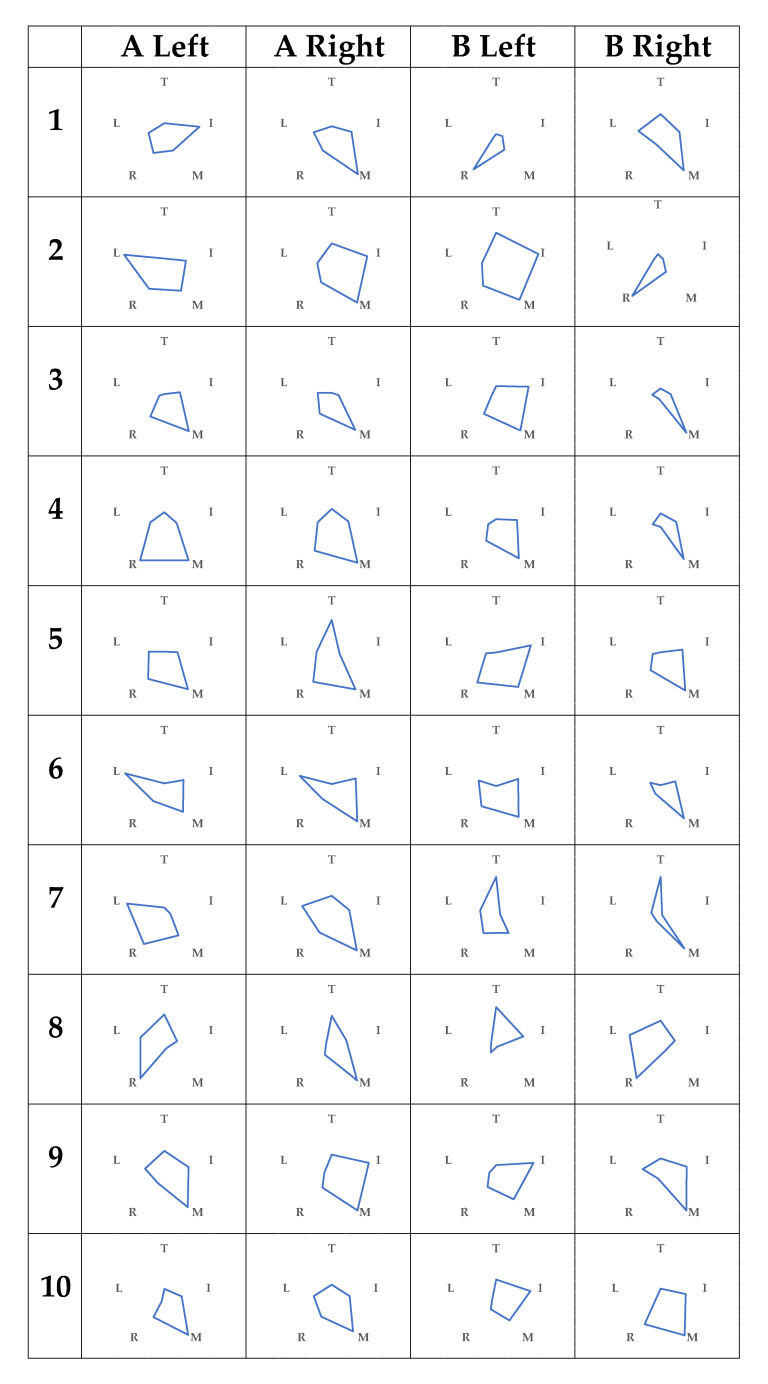
Radar charts of raw contact-force data.

The relationship between the raw contact-force data and condition 1 was superimposed; therefore, it was not easy to understand the fingers’ relationship. Figure 5 shows radar charts of the raw contact-force data.

Overall, the raw contact-force data for the middle and occasional ring finger were large. In addition, there was a tendency that the variation of the finger was small from condition 6 onwards.

### 3.2. Number of Times to Grip and Release

Table 4 shows the combinations with the highest matching rate without error in the number of grip and release and the single item with the highest matching rate. The right column shows the grip-and-release conditions for different numbers of times.

The highest matching rate was 80.0%. Both combinations included the z-axis acceleration of the middle finger and the raw contact-force data of the little finger. The highest single matching rate was 75.0% of the z-axis acceleration of the middle finger. Conditions 5, 9, and 10 were often wrong. The left hand made more errors than the right hand.

### 3.3. Frequency Characteristics

**Figure 6:**
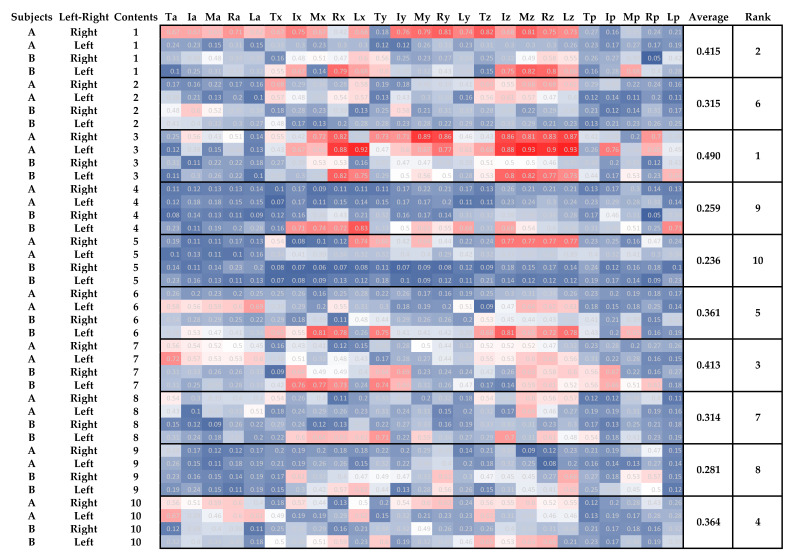
Frequency analysis result: The value of the highest power frequency divided by the total power and the average value and its ranking by condition.

Figure 6 shows the results of the frequency analysis, where the highest frequency one divided by the total power is shown on a scale of red for the value close to 1 and blue for the value close to 0.

The mean was greater than 40% in conditions 1, 3 and 7. However, the mean was lower than 30% in conditions 4, 5, and 9.

### 3.4. Correlation of Each Finger

**Figure 7:**
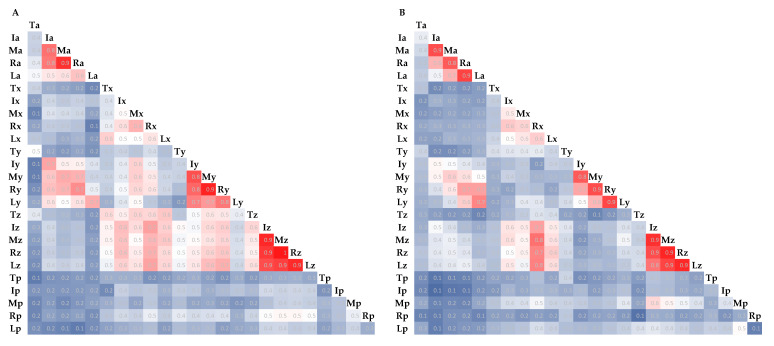
Mean of the correlation coefficients for each of conditions 1 to 5 (**A**: subject A, **B**: subject B).

Conditions 1 to 5 were different, but the point at which all fingers moved simultaneously was the same. Therefore, after calculating the correlation coefficients for each item of data for each finger, the mean values were calculated for each subject. The value close to 1 is shown in red, and the value close to 0 is shown in blue on a scale in Figure 7.

In common with the subjects, the correlations were high for all combinations of the index and middle finger combined acceleration, index and middle finger y-axis acceleration, middle and ring finger y-axis acceleration, ring and little finger y-axis acceleration, and index finger to little finger z-axis acceleration. The combination with the highest mean of correlation coefficients was the z-axis acceleration of the middle finger and the z-axis acceleration of the ring finger for both subjects. The combination with the overall thumb item and raw contact-force data had a low correlation. 

**Figure 8:**
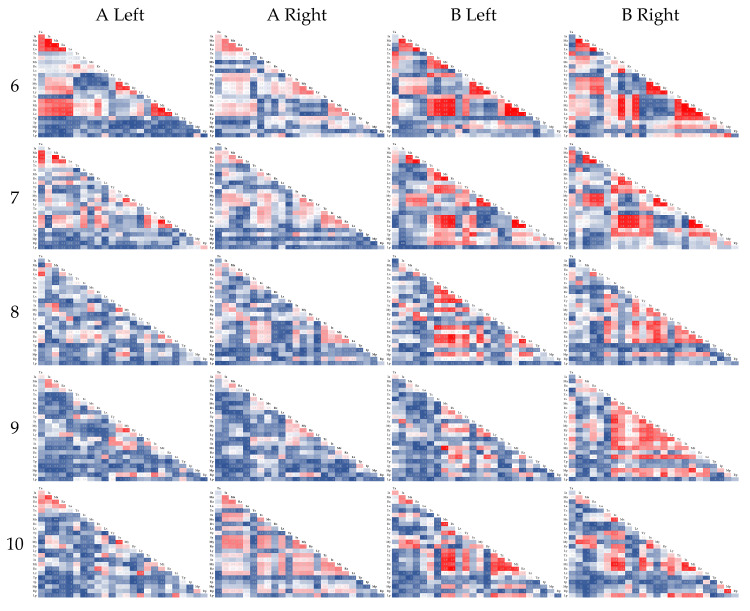
Correlation coefficient after condition 6.

The mean of the correlation coefficients for each item from condition 6 onwards, when no specific finger is moved, is shown in Figure 8. Values close to 1 are shown as red and values close to 0 are shown as blue on a scale.

Compared with Figure 7, the relationship between the index, middle, and ring fingers tended to be stronger in condition 6; after 7, it tended to be weaker. Subject A had a particularly low correlation. There was also a high correlation between subject A’s right hand in condition 10. Subject B correlated highly with the right hand in condition 9.

## 4. Discussion

### 4.1. Data Variation

A total of 25 combinations were shown each finger and each item. From Table 3, regarding the significant differences between the left and right hands, A and B showed significant differences in 24% and 16% of the combinations, respectively, but the differences were not considered significant overall. Regarding the significant differences between subjects, significant differences were found in 32% of the combinations for the right hand and 36% for the left hand, but no significant differences were considered overall. However, the differences in raw contact-force data were particularly large between subjects. We need further validation of the data for healthy subjects.

In Figure 3, Figure 4 and Figure 5, we found that the use of radar charts helps us to intuitively understand how the grip and release of radar charts occur. In particular, since absolute acceleration is an index of momentum, the variability of the whole of absolute acceleration was smaller in the case of condition 3. In addition, in the case of condition 6 and later, the variability of the absolute acceleration of the corresponding finger was small for both subjects. The z-axis acceleration was also considered to have a similar tendency because the grip and release of the fingers can be considered a vertical motion. Figure 5 shows that the same tendency was observed in the raw contact-force data because of the finger distortion in the gripping motion. In the raw contact-force data, the raw contact-force data of the middle and ring fingers were also larger, which was thought to be because these fingers were longer in hand and were more in contact with the palm when gripping.

In comparison with the actual grip and release of subject A in Figure 3, the variability of the x-axis acceleration (left–right) in condition 1 tended to be large in both the left and right hands and was considered because subject A grasps with her middle finger when she naturally grasps the hand. By using the radar charts, we found that the grip-and-release speed of condition 2, which assumed of a 10-s grip and release, was different from that of condition 1, but the motion itself did not change much from that of condition 1 (only the tempo of grip was changed). In condition 5, the radar charts did not capture the tremor, but it was found that she gripped and released her hands less and spread her hands more often. As for subject B in Figure 4, from the actual conditions and the variation of the x-axis acceleration, it was found that the middle finger gripped and released without any side-to-side motion. Moreover, unlike subject A, the grip and release of condition 2, which was simulated as 10-s grip and release, was more intense than in condition 1.

Thus, it is possible to grasp the trend from the radar charts without checking the grip and release of the radar. Medical professionals and the public were to be used to elucidate the abnormality of the hand and to check the progress of rehabilitation. However, it is not possible to obtain grip and release under different conditions for the same person, making it necessary to build a model for healthy people.

### 4.2. Number of Times to Grip and Release

Table 4 shows that the combinations with a high matching rate include the z-axis acceleration of the middle finger. Moreover, the matching rate increased when the raw contact-force data of the middle finger were combined with the raw contact-force data, not the z-axis of the middle finger alone, and since we considered the raw contact-force data in 4.1 as well, it was thought that obtaining information on strain is effective. Although the error was larger for certain conditions, such as conditions 5 and 9, the number of times the error was calculated using an abnormal grip estimated by combining it with other evaluations.

### 4.3. Frequency Characteristics

As in Section 4.1, there were 25 combinations for each finger and each item. The larger the power of the most potent frequency divided by the total power, the more periodicity is considered present. As shown in Figure 6, conditions 1, 3, and 7, which had higher values, were also cyclic in terms of actual grip and release. However, conditions 4, 5, and 9, where the mean value was below 30%, showed little periodicity in the grip-and-release movements. In the case of condition 4, the values were smaller because of the irregular grip-and-release instructions. In the case of condition 5, where the user was instructed to grip and release while trembling, the periodicity of the trembling was considered the noise, and values were smaller. Moreover, under condition 9, where the ring finger was instructed not to move, the high correlation between the middle and ring fingers affected the grip-and-release movements (Figure 6), which will be discussed in 4.4. Based on these results, it was considered possible to detect anomalies in the periodicity of grip and release by dividing the power of the most potent frequency divided by the total power.

### 4.4. Correlation of Each Finger

As in Section 4.1, there were 25 combinations for each finger and each item. Figure 7 shows that the high correlation from the index finger to the little finger was considered due to the connection of the tendons used to move the fingers. In particular, the middle and ring fingers were interlocked. The z-axis acceleration of the middle and ring fingers was also included in the combinations with the highest accuracy in Table 4. That is, the combination of the z-axis acceleration of the middle finger and the z-axis acceleration of the ring finger is considered to accentuate the grip-and-release features. The reason for the low correlation of the combinations of thumb data was due to the lack of motion during the grip-and-release motions and the fact that the motions are more independent than those of other fingers. Thus, we thought it is possible to consider the anatomical structure of the hand concerning to the correlation. After condition 7 in Figure 8, the correlation tended to be lower from the index finger to the little finger, and the right hand of subject A in condition 9 was particularly visually abnormal. For example, if a patient has this condition, regular measurement of his or her condition during rehabilitation would help visualize and quantify the progress of rehabilitation.

From the above, it was considered possible to understand the characteristics of grip and release and the state of the hand by combining each method. It was also thought that visualization on radar charts and scales would allow for an intuitive understanding of the features. However, since the subjects were healthy women and not all hand movements were covered, the generalization of the results is limited.

## 5. Limitations

The number of subjects is small, and the number of data is small. Our evaluation method’s usefulness was suggested, and we will obtain more data, including the presence or absence of diseases. The number of N required for each disease will be determined by conducting a usefulness evaluation. Specifically, 10 cases of data for each disease should be accumulated, and the number of Ns should be determined by evaluating significant differences (including ROC curves). If it is difficult to distinguish from other diseases, it is necessary to accumulate the number of Ns over time.

The sensors used in this study proved to be useful in providing strain information. However, they were sometimes difficult to attach and challenging to operate during the experiments; therefore, it is necessary to investigate or develop a smaller, lighter, and easier attach the sensor. IN addition, the use of wireless systems is expected to be more widespread.

## 6. Conclusions

In this study, we used an accelerometer and a strain sensor to acquire data on the hand grip-and-release movements during a 10-s grip and release. Feature extraction of each finger, calculation of the number of times to grip and release the data, frequency analysis, and correlation between each finger were derived. Different characteristics were obtained depending on the grip-and-release patterns. It was suggested that the state of grip and release of the radar charts were intuitively known by expressing it in the radar charts. The raw contact-force data were found to be useful for knowing the characteristics of grip and release and for improving the accuracy of calculating the number of times of grip and release. Frequency analysis suggests that knowledge of the periodicity of grip and release can detect unnatural grip and release and tremor states. The correlations between the fingers allowed us to consider the grip-and-release characteristics of the fingers, considering the anatomy of the hand. By taking these factors into account, it was thought that the 10-s grip-and-release test could be given a new value by objectively assessing the motor functions of the hands, other than the number of times of grip and release.

However, this study was a proof-of-concept study, and the subjects were two healthy females. There are limitations in generalizing the results of this experiment and using them in clinical practice. In future studies, we will collect and analyze data from a larger number of subjects and diseases to establish the evaluation suggested in this study as useful.

## Figures and Tables

**Table 1:** Contents of the data output by HapLog.

CSV File	
Header information	Version information, timestamp, and memo
Sensor information (for each sensor)	Position of a sensor in connector, name, unit of target data, number of data, and the maximum value and minimum value
Measurement data	Time, finger contact force, absolute acceleration, raw contact-force data (με), x-axis acceleration, y-axis acceleration, z-axis acceleration, and mark

**Table 2:** The label each item on each finger.

Label	
Finger	T (thumb), I (index finger), M (middle finger), R (ring finger), and L (little finger)
Item	a (Absolute Acceleration), x (x-axis acceleration), y (y-axis acceleration), and z (z-axis acceleration) p (raw contact-force data)

**Table 3:** Results of the significance difference test (left–right by subject, each subject is by right and left).

Significant Difference (*p* < 0.05)	
left–right	Subject A: Mx, Rx, Ry, Rz, La, Lx Subject B: Ix, Mp, Rx, Lp
subject A–subject B	Right: Tp, Iz, Ip, Mx, Mp, Rx, Lz, Lp Left: Tz, Tp, Ix, Iz, Ip, Mx, Rx, Rz, Rp,

**Table 4:** Combination and number of grip and release.

Combination	Accuracy	Conditions with Different Number of Grip and Release: Subjects, Hands, and Conditions Performed (Error)
TyMzRzRpLp	80.0%	ARight9(−6), ALeft2(3), ALeft8(1), ALeft9(−1), ALeft10(−2) BLeft 2(2), BLeft 5(−4), BLeft 8(−1)
MzMpLp	80.0%	ARight6(1), ARight9(−9), ALeft1(−1), ALeft9(-2), ALeft10(−1) BRight 5(−8), BRight 8(3), BLeft5(−3)
Mz	75.0%	ARight4(1), ARight5(−1), ARight6(1), ARight9(−9), ALeft1(−1), ALeft10(−1) BRight2(−2), BRight5(−8), BRight8(2), BLeft5(−4)

## Data Availability

Date sharing not applicable.
